# Whole-transcriptome sequencing uncovers core regulatory modules and gene signatures of human fetal growth restriction

**DOI:** 10.1186/s40169-020-0259-0

**Published:** 2020-01-28

**Authors:** Guiying Wang, Jun Yu, Yiwei Yang, Xiaoqin Liu, Xiaobo Zhao, Xudong Guo, Tao Duan, Chenqi Lu, Jiuhong Kang

**Affiliations:** 1 Clinical and Translational Research Center of Shanghai First Maternity and Infant Hospital, Shanghai Key Laboratory of Signaling and Disease Research, School of Life Sciences and Technology, Tongji University, Shanghai, China; 2 Department of Biostatistics and Computational Biology, State Key Laboratory of Genetic Engineering, School of Life Sciences, Fudan University, Shanghai, China; 3 Shanghai First Maternity and Infant Hospital, Tongji University School of Medicine, Shanghai, China

**Keywords:** Fetal growth restriction, Case–control study, Whole-transcriptome, lncRNAs, miRNAs, Gene co-expression network

## Abstract

**Background:**

Fetal growth restriction (FGR) contributes the primary cause of perinatal mortality and morbidity with impacts on the long-term health. To determine the core gene expression network and gene signatures, which in combination with ultrasound confirmation will more effectively differentiate constitutionally normal small for gestational age and pathological FGR groups, we performed RNA sequencing for protein-coding genes, lncRNAs, and small RNAs in a case–control study of umbilical cord blood.

**Results:**

Five pairs of FGR case and control umbilical cord blood samples were used for RNA sequencing and weighted gene co-expression network analysis (WGCNA). Results showed that 339 mRNAs, 295 lncRNAs, and 13 miRNAs were significantly differentially expressed between FGR cases and controls. Bioinformatics analysis indicated that these differentially expressed molecules were mainly involved in metabolism, neural, cardiac, and immune systems, and identified 18 WGCNA modules for FGR. Further quantitative verification was performed using umbilical cord blood and maternal peripheral blood from 12 pairs of FGR cases and controls. The logistic regression and receiver operating characteristic curve indicated that RP11_552M6.1, LINC01291, and Asgr1 in umbilical cord blood, while Sfrp2, miR-432-5p, and miR-1306-3p in maternal peripheral blood had potential significance for FGR.

**Conclusions:**

We comprehensively profiled the whole-transcriptome landscape of human umbilical cord blood with FGR, constructed the core WGCNA modules, and delineated the critical gene signatures of FGR. These findings provide key insight into intrauterine perturbations and candidate signatures for FGR.

## Background

Fetal growth restriction (FGR) is a common pregnancy complication that occurs in 5–10% of all pregnancies. FGR is the primary cause of perinatal mortality and morbidity, and has impacts on the long-term health of the offspring [1–3]. Children surviving FGR are at a greater risk of developing neurodevelopmental dysfunction during childhood and cardiovascular and/or metabolic diseases subsequently in life [4]. The increased risk of cardiovascular disease is due to the increased workload of the fetal heart, which is associated with postsystolic shortening and fetal exposure to higher levels of maternal cortisol [5]. Additionally, individuals with FGR have a higher tendency to develop obesity [6]. Preterm delivery is a major strategy for resolving this problem but may result in a nonviable fetus [7, 8]. Late-onset FGR, which occurs after 32 weeks of gestation, is more complicated, results in fewer characteristic histological changes and has an unknown underlying mechanism [9]. Thus, timely and accurate prenatal detection of FGR remains challenging.

Recent developments in high-throughput sequencing enable the assessment of the entire transcriptome of mRNAs, long noncoding RNAs (lncRNAs), and microRNAs (miRNAs) with the potential to uncover the biological processes driving complex phenotypes. Systems biology methods can better capture the complexity of inter-gene relationships and the signaling pathways associated with diseases, and offer the opportunity to better define the co-regulatory patterns that underlie complex phenotypes. Weighted gene co-expression network analysis (WGCNA) has been successfully applied in several studies to facilitate the systems-level characterization of expression pattern by clustering highly-correlated genes into co-expression modules with conserved biological functions [10, 11].

Studies assessing the transcriptome-wide profile of human placentas with FGR are beginning to emerge, and a few protein-coding genes and noncoding RNAs have been assessed [12, 13]. However, the inconsistent findings suggest that placental biomarkers have a low reliability, limiting their clinical ability [14]. FGR, especially abnormal fetal growth confirmed by repeated ultrasound, has a multifactorial nature with many causes, including maternal, fetal, and placental factors [15]. Birth weight is correlated with the maternal body mass index (BMI) and delivery gestational age [16]. Moreover, information from previous studies is limited by sample heterogeneity, such as placental differences, and a focus on univariate gene expression analyses contrasting normal and adverse phenotypic outcomes. Thus, a case–control study with a matching control to each FGR infant, could eliminate the confounding factors and provide more persuasive evidences. Furthermore, high-throughput sequencing for the entire transcriptome of umbilical cord blood as the origin of FGR infant could provide an exemplary opportunity to demonstrate the core gene networks by elucidating fetal growth-related processes.

Therefore, in combination with the core gene expression network obtained from a case–control study of umbilical cord blood, the ultrasound confirmation could more effectively differentiate constitutionally normal small for gestational age and pathological FGR groups, and provide candidate approaches for disease intervention and prevention at an early time-point. In this study, we comprehensively profiled the transcriptome-wide landscape of human umbilical cord blood in a case–control study by implementing a network-based approach to construct the core gene co-expression network and delineate the critical gene signature of FGR.

## Methods

### Study participants

Using a case–control study, FGR cases and corresponding controls were matched according to gestational age, maternal BMI and age (Additional file 1: Table S1) to exclude the maternal factors. The inclusion criteria of FGR were based on the birth weight reference percentiles as an estimated weight below the 10th percentile for gestational age [17]. These FGR and control infants had a birth score not less than 9 and no birth defects. The women had no smoking history and no other pregnancy complication, and the women with preeclampsia and other complications of pregnancy were excluded. Five pairs of FGR cases and controls obtained at Shanghai First Maternity and Infant Hospital (Tongji University, Shanghai, China) between 2017 and 2018 were used for RNA sequencing. Further quantitative RT-PCR verification was performed in the umbilical cord blood and maternal peripheral blood obtained from the 12 FGR cases and 12 controls (Additional file 2: Table S2). All women provided written informed consent, and the study protocol was approved by the Ethics Committee of Shanghai First Maternity and Infant Hospital (No. KS17115).

### Sample collection

In total, 2.5 mL of umbilical cord blood and 2.5 mL of maternal peripheral blood were collected at the time of delivery into PAXgene whole blood RNA tubes (PreAnalytix) and stored at 25 °C for at least 2 h, at − 20 °C for 24 h, and at − 80 °C until processing. The total RNA was extracted using a RNeasy Protect Animal Blood Kit (Qiagen) according to the manufacturer’s instructions. The RNA concentration and purity were measured using a NanoDrop ND100 spectrophotometer (Thermo Scientific) and BioAnalyzer 2100 system (Agilent).

### RNA-sequencing workflow

For lncRNAs and mRNA, the RNA-sequencing library generation, workflow, and data analysis were performed as previously described [18]. The small RNA sequencing including miRNAs was also performed. After the automatic quality control and adapter trimming by Trim_Galore (http://www.bioinformatics.babraham.ac.uk/projects/trim_galore/), the RNA paired-end reads were mapped to the human genome hg38 by HISAT2 [19], and quality controlled using RSeQC [20]. Based on the annotation file (Homo_sapiens.GRCh38.83.gtf) in the Ensembl database, the read counts were calculated by featureCounts [21], and normalized by rlogTransformation. The differentially expressed genes (DEGs) were analyzed by DESeq 2 package in R [22].

### Bioinformatics and stability analyses

The lncRNA targets were predicted with LncTar, RNAplex, and RIsearch, while the miRNA targets were predicted with miRanda, PITA, and RNAhybrid. The gene ontology (GO) and signaling pathway enrichment analyses were completed using DAVID web servers [23]. Based on the log_2_(Fold Change), we performed the gene set enrichment analysis (GSEA) for the significant signaling pathways and imprinted genes [24–26]. The gene co-expression network was generated using the WGCNA package in R [11]. The parameters were as follows: networkType = unsigned, corType = Pearson, Power = 9, minModuleSize = 50, mergeCutHeight = 0.35, reassignThreshold = 0.99 and the remaining default parameters. The regulatory network was illustrated by the igraph package in R, and a circos graph was obtained by the RCircos package [27]. The logistic regression analysis and receiver operating characteristic (ROC) curve were performed by glm and pROC packages, and 95% CI of area under the curve (AUC) were calculated by boot package in R. The stability of gene modules was also assessed [28].

### Quantitative RT-PCR analyses

The first-strand cDNA was synthesized by M-MLV Reverse Transcriptase (TaKaRa) with 500 ng total RNA. The qRT-PCR assays were performed with SYBR^®^ Premix Ex TaqTM II (TaKaRa) on an Mx3000P QPCR System (Agilent). The qRT-PCR primers for the candidate protein-coding genes and lncRNAs were shown in Additional file 3: Table S3. The Bulge-LoopTM miRNA qPCR Primer Sets (RiboBio) were used for the expression detection of miRNAs. The expression levels of protein-coding genes and lncRNAs were normalized to GAPDH, while the miRNAs were normalized to U6. The statistical significance was analyzed by Student’s *t*-test.

## Results

### Significantly different transcriptome of FGR and control umbilical cord blood

**Fig. 1:**
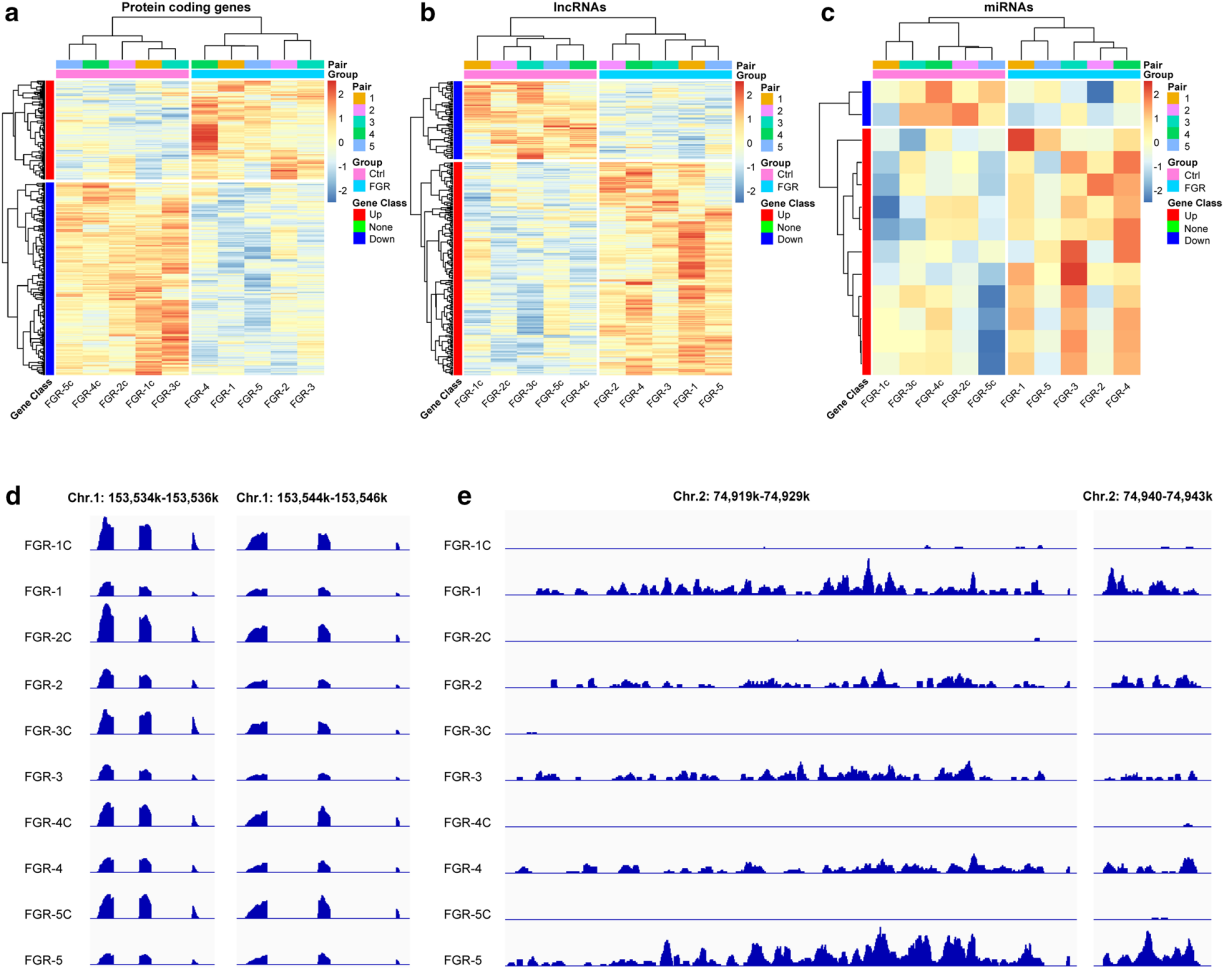
Significantly different transcriptome of FGR and control umbilical cord blood. **a**–**c** Hierarchical clustering analysis identified 339 protein coding genes (**a**), 295 lncRNAs (**b**), and 13 miRNAs (**c**), that are differentially expressed between FGR and the corresponding control. **d** Examples of critical protein coding genes (S100A6, S100A4) expressed in normal control that is repressed in FGR. **e** Examples of critical lncRNAs (LINC01291, LINC01293) not expressed in normal control that becomes activated in FGR

To examine differences in the entire transcriptome between FGR cases and normal controls, we performed RNA sequencing for protein-coding genes, lncRNAs, and miRNAs in a case–control study with five pairs of umbilical cord blood samples (Additional file 1: Table S1). The differential expression analysis and hierarchical clustering showed that the protein-coding genes mostly tended to be down-regulated (Fig. 1a), while the lncRNAs (Fig. 1b) and miRNAs (Fig. 1c) tended to be up-regulated in the FGR cases as compared to the corresponding controls. All of the differentially expressed genes including protein coding genes, lncRNAs, and miRNAs were shown in Additional file 4: Table S4. The representative differentially expressed protein coding genes and lncRNAs were shown in Fig. 1d, e, respectively. These findings indicate that the entire transcriptome of umbilical cord blood from FGR case is significantly different from the corresponding control.

### Differentially expressed protein-coding genes and physiological functional signaling pathways

**Fig. 2:**
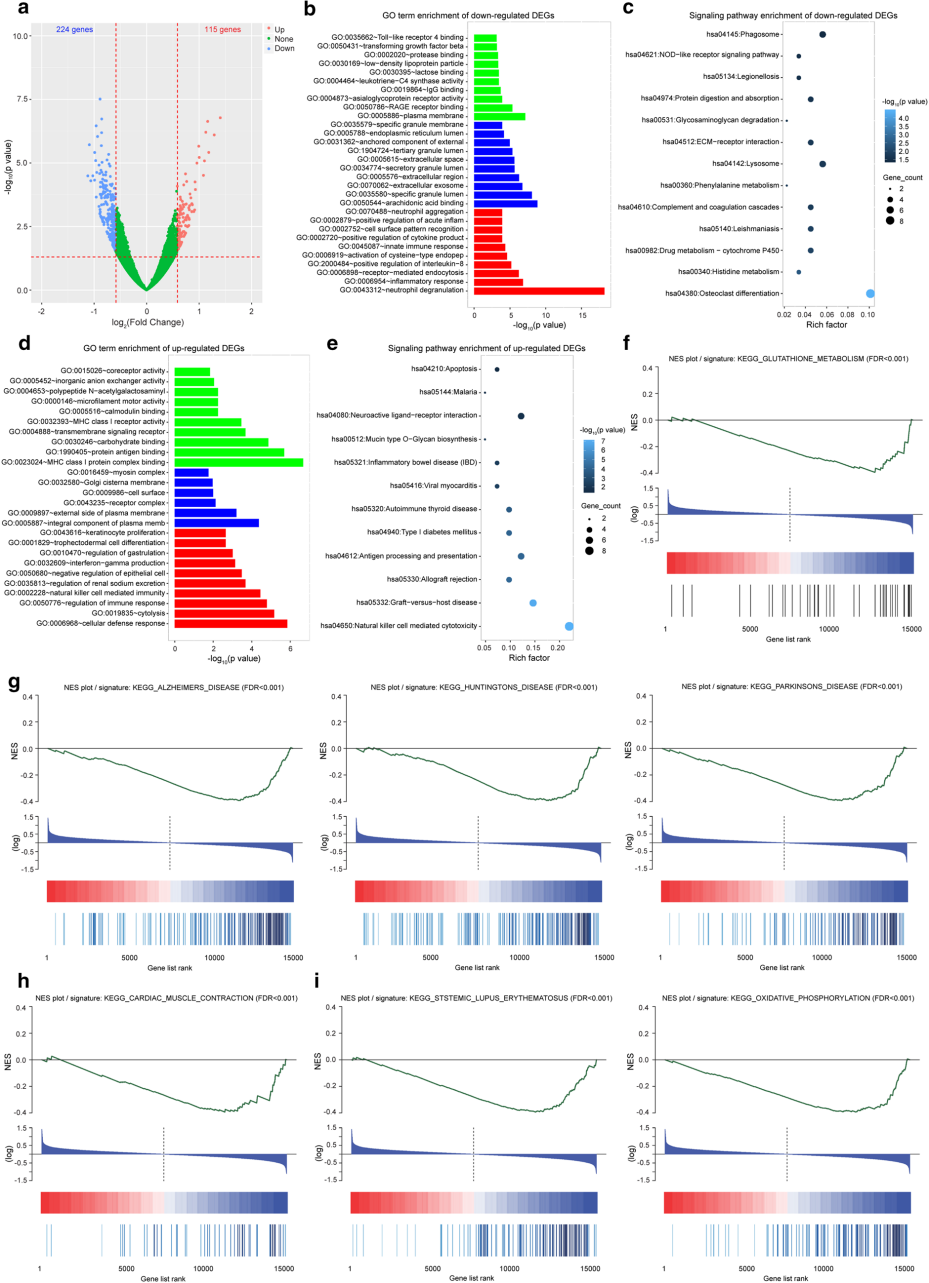
Differentially expressed protein-coding genes and physiological functional signaling pathways. **a** Volcano plot of the differentially expressed protein-coding genes (115 up-regulated and 224 down-regulated). **b** GO term enrichment of the down-regulated differentially expressed protein-coding genes. Green, molecular function. Blue, cellular component. Red, biological process. **c** Signaling pathway enrichment of the down-regulated differentially expressed protein-coding genes. **d** GO term enrichment of the up-regulated differentially expressed protein-coding genes. **e** Signaling pathway enrichment of the up-regulated differentially expressed protein-coding genes. **f**–**h** GSEA plots of FGR-related adult diseases, including metabolism (**f**), neural system (**g**), and cardiac system (**h**). **i** GSEA plots of predictive FGR-related diseases, including immune system (left panel) and oxidative phosphorylation (right panel)

Among the 339 differentially expressed protein-coding genes, 224 genes were down-regulated, and 115 genes were up-regulated in FGR cases (Fig. 2a, Additional file 4: Table S4). The GO enrichment analysis showed that these down-regulated genes were mainly enriched in plasma membrane, arachidonic acid binding, and neutrophil degranulation (Fig. 2b). Further signaling pathway analysis indicated that these genes were mainly involved in osteoclast differentiation, phagosome, and lysosome (Fig. 2c). Additionally, the up-regulated genes were mainly enriched in MHC class I protein complex binding, the cellular defense response (Fig. 2d), natural killer cell-mediated cytotoxicity and Graft-versus-host disease (Fig. 2e). Further GSEA results (Additional file 5: Table S5, Additional file 6: Table S6, Additional file 7: Table S7) showed that these genes were mainly enriched in glutathione metabolism (Fig. 2f), Alzheimer’s disease, Parkinson’s disease, Huntington’s disease (Fig. 2g), cardiac muscle contraction (Fig. 2h), systemic lupus erythematosus and oxidative phosphorylation (Fig. 2i). These findings indicate that the differentially expressed protein-coding genes are not only enriched in known FGR-related processes, such as metabolism and neural and cardiac systems, but also significantly associated with the immune system.

### Differentially expressed miRNAs and physiological functional signaling pathways

**Fig. 3:**
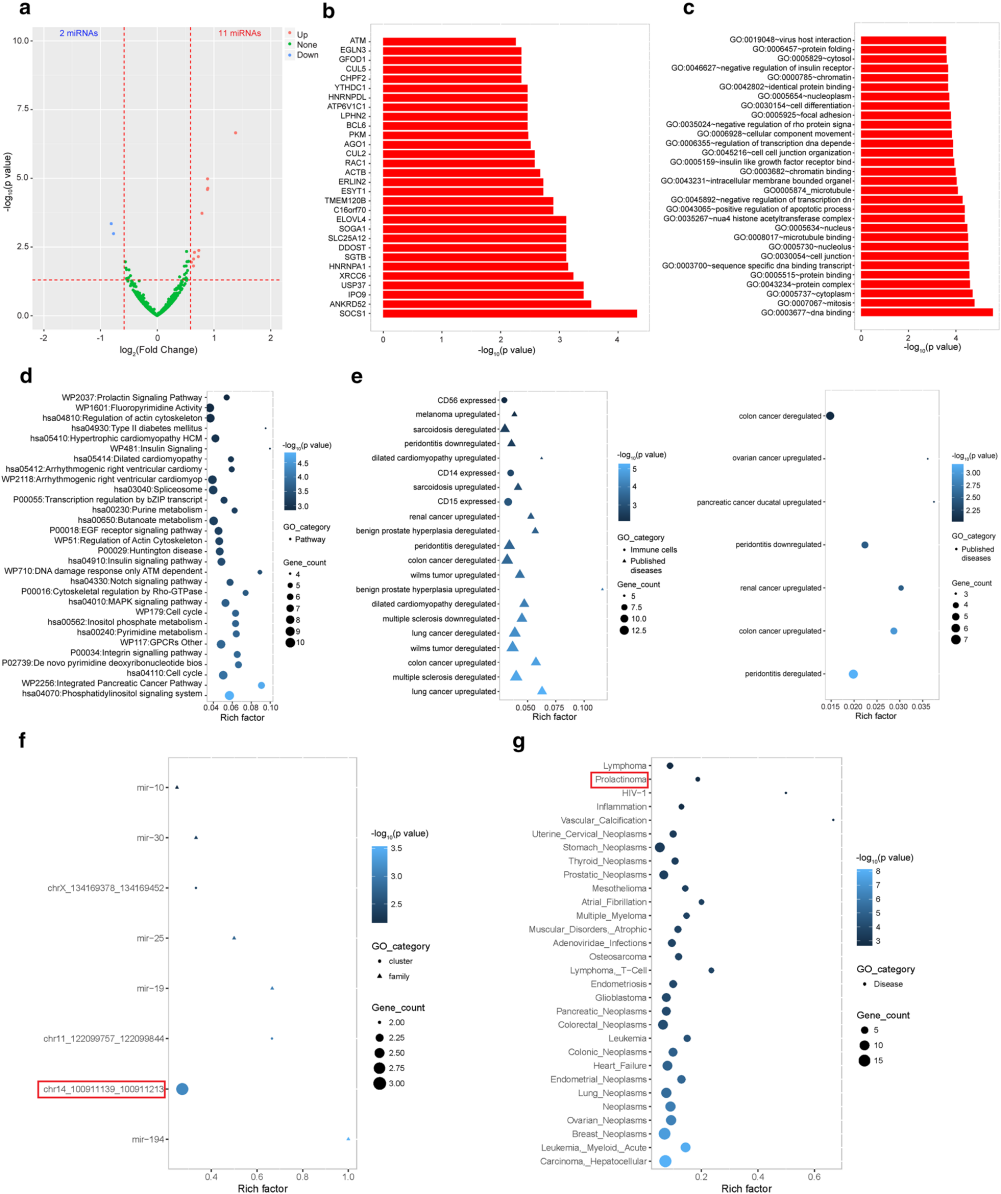
Differentially expressed miRNAs and physiological functional signaling pathways. **a** Volcano plot of the differentially expressed miRNAs (11 up-regulated and 2 down-regulated). **b** The top-30 popular targets as predicted for the critical miRNAs (top-30). **c** GO enrichment of the predictive targets of the critical miRNAs (top-30). **d** Signaling pathway enrichment of the predictive targets of the critical miRNAs (top-30). **e** Specific diseases in TCGA database correlated with the predictive targets of the critical miRNAs (left panel, top-30; right panel, 13 differentially expressed miRNAs). **f** Clustering and family analyses of the precursors of the critical miRNAs (top-30, red for 13 differentially expressed miRNAs). **g** Specific diseases correlated with the precursors of the critical miRNAs (top-30, red for 13 differentially expressed miRNAs)

Based on the top-30 highly varied miRNAs with larger log_2_(Fold Change) including the 13 significantly differentially expressed miRNAs (Fig. 3a, Additional file 4: Table S4), we predicted miRNA targets by miRanda, PITA, and RNAhybrid. The top-30 popular target genes as regulated by these miRNAs were shown in Fig. 3b, including the suppressor of cytokine signaling 1 (SOCS1), which is required for normal postnatal growth and survival [29, 30]. Further GO (Fig. 3c) and signaling pathway (Fig. 3d) analyses showed that the predicted targets of these miRNAs mainly focused on DNA binding, mitosis, integrated pancreatic cancer pathway, DNA damage response only ATM dependent, insulin signaling, and type II diabetes mellitus. An integrated analysis of the predicted targets and human diseases using the Human MicroRNA Disease Database (HMDD, v2.0) indicated that these miRNAs were significantly correlated with immune cells and published diseases (Fig. 3e). The clustering and family analysis showed that these miRNA precursors focused on the miR-194 family and Chr14_100911139-100911213 cluster (Fig. 3f), and neoplasm, leukemia, inflammation, and prolactinoma (Fig. 3g). For the 13 significantly differentially expressed miRNAs, we also observed the significance of the Chr14_100911139-100911213 cluster and prolactinoma. These findings indicate that the significantly varied miRNAs are not only involved in FGR-related diseases but also focused on specific gene clusters and various diseases.

### Core regulatory network and imprinted genes of FGR

**Fig. 4:**
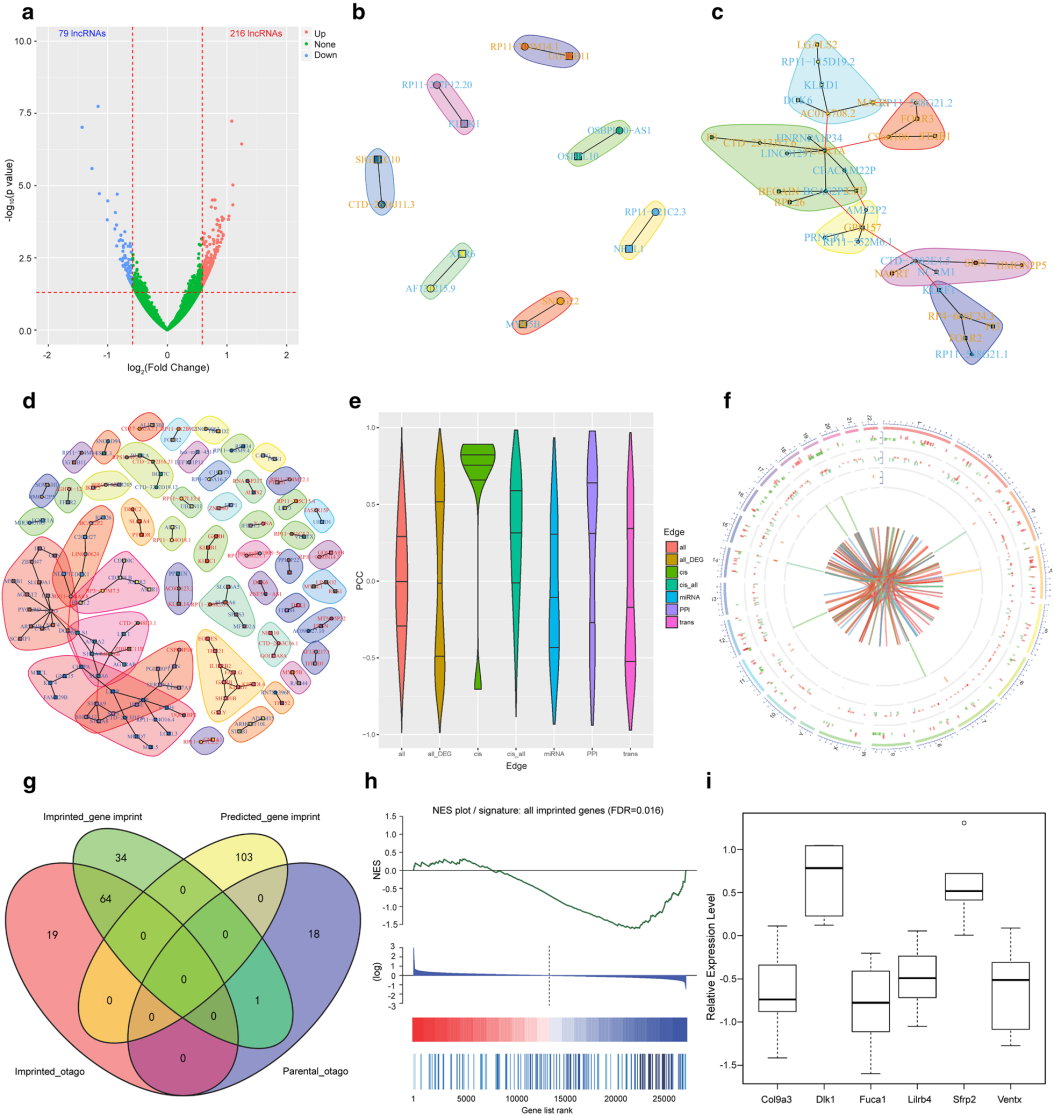
Core regulatory network and significant imprinted genes of FGR. **a** Volcano plot of the differentially expressed lncRNAs (216 up-regulated and 79 down-regulated). **b** Predicted *cis*-regulatory relationships among the differentially expressed protein-coding genes and lncRNAs. **c** Representative *trans*-regulatory relationships among the top 35 differentially expressed protein-coding gene and lncRNAs. **d** Regulatory network among the differentially expressed protein-coding genes, lncRNAs, and miRNAs after screening with the correlation coefficient (> 0.9). **e** PCC plots of all differentially expressed lncRNAs, protein-coding genes, and miRNAs. DEG, differentially expressed genes. PPI, protein–protein interaction. **f** Circos plot of all differentially expressed protein-coding genes, lncRNAs, and miRNAs on human chromosomes. **g** Imprinted genes collected from the Gene imprint and Otago databases. **h** GSEA plot of all imprinted genes in FGR and control umbilical cord blood samples. **i** Six imprinted genes (Col9a3, Dlk1, Fuca1, Lilrb4, Sfrp2, and Ventx) significantly differentially expressed in umbilical cord blood of FGR versus control

To systematically investigate the core regulatory network of FGR at the entire transcriptome level, we performed RNA sequencing for the expression profile of lncRNAs, and found 79 lncRNAs significantly down-regulated and 216 lncRNAs significantly up-regulated (Fig. 4a, Additional file 4: Table S4). Further analyses of the regulatory relationship among the 339 protein-coding genes and 295 lncRNAs showed that seven lncRNAs were predicted to *cis*-regulate their neighboring protein-coding genes within a 2-kb region (Fig. 4b), and 7616 *trans*-regulatory relationships were obtained. To clearly demonstrate the *trans*-regulatory relationships, the top-35 significantly differentially expressed lncRNAs and protein-coding genes with larger degrees was shown (Fig. 4c). In combination of lncRNA-mRNA, miRNA-mRNA, and miRNA-lncRNA analyses, we observed 7 *cis*-, 59 *trans*-, and 2 miRNA regulatory relationships after screening with the Pearson correlation coefficient (PCC) (absolute value > 0.9) (Fig. 4d). The lncRNAs and protein-coding genes in *cis*-regulatory relationships had a positive PCC, while the lncRNAs and their *trans*-regulated protein-coding genes had a negative PCC (Fig. 4e). All significantly differentially expressed molecules and the interactions were shown in the circos plot (Fig. 4f).

As imprinted genes are critical in growth and development [31], we performed further analysis based on 240 imprinted genes obtained from two existed public databases (http://www.geneimprint.com and http://igc.otago.ac.nz/home.html) (Fig. 4g). The expression levels of these imprinted genes were significantly negatively associated with FGR (Fig. 4h), and six imprinted genes (Col9a3, Dlk1, Fuca1, Lilrb4, Sfrp2, and Ventx) were significantly differentially expressed between FGR cases and controls (Fig. 4i). These findings observed a cluster of imprinted genes as correlated with FGR and might provide potential signatures for FGR.

### Critical gene co-expression network modules closely correlated with FGR

**Fig. 5:**
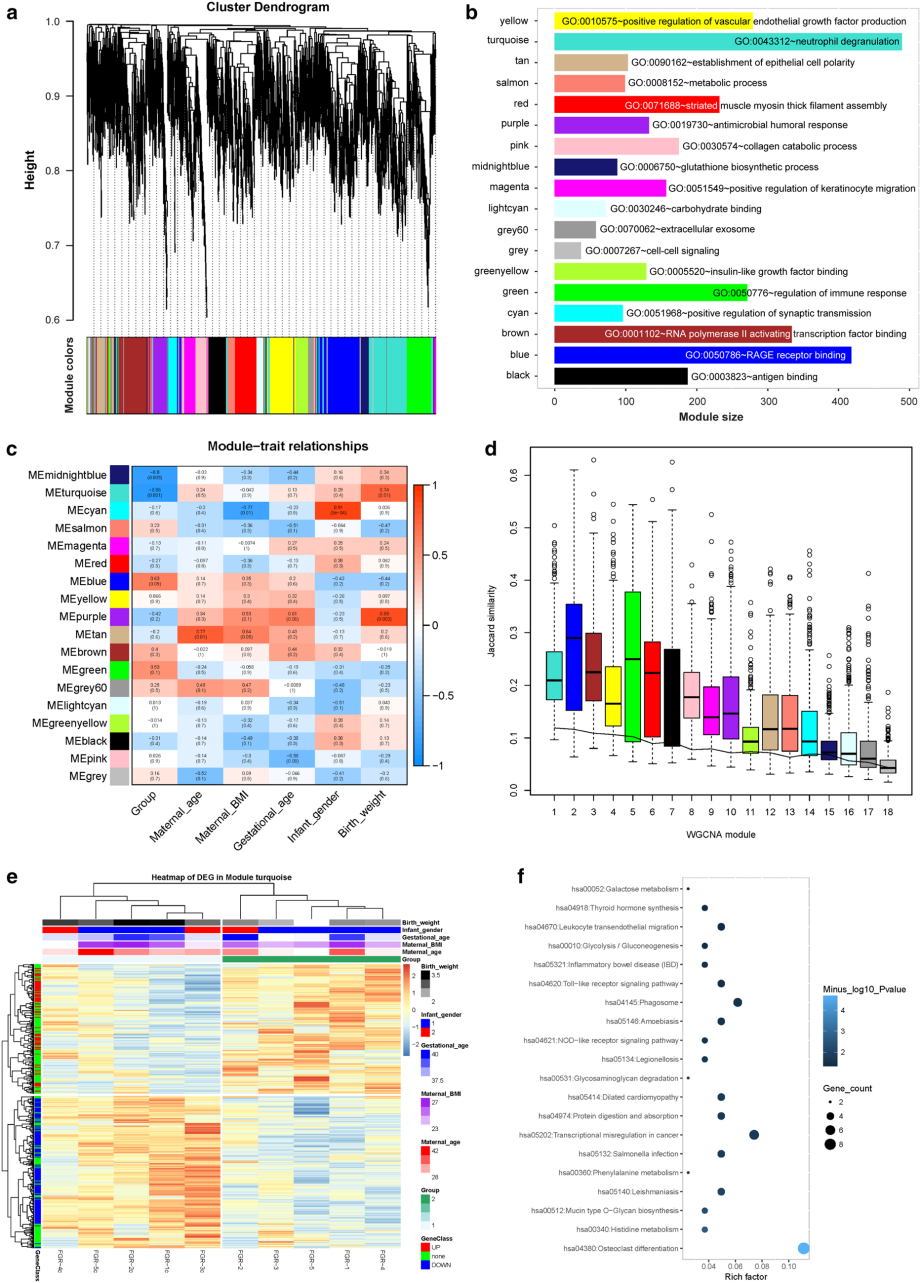
Critical gene co-expression network modules closely correlated with FGR. **a** Results of the WGCNA and clustering of the entire transcriptome. **b** Module size of the gene co-expression network and GO enrichment analyses of 18 modules. **c** Person correlation analysis of the network modules and continuous demographic characteristics of the FGR infants. The color gradient indicates the direction, i.e., positive (red) and negative (blue), and the strength of the correlation. **d** Boxplot of stability (Jaccard similarity coefficient) between each module of real data and modules from 1000 bootstrap re-sampling datasets. The line indexes the best-case stability of the random modules in the simulation. **e** Hierarchical clustering of candidate genes in the representative turquoise module. **f** Signaling pathway analysis of the turquoise module

To clarify the significant gene co-expression network involved in FGR, we performed WGCNA and clustered the entire transcriptome of FGR cases and controls (Fig. 5a). All genes focused on 18 modules (Additional file 8: Table S8), and most genes were clustered in the turquoise module as enriched in neutrophil degranulation (Fig. 5b). The module salmon was mainly enriched in metabolic process, while module green focused on regulation of immune response. The Pearson correlation analysis of the relationships between the network modules and sample characteristics showed that the turquoise and purple modules were significantly positively correlated with birth weight, while the turquoise and midnightblue modules were significantly different between FGR cases and controls (Fig. 5c). Similarly, we found that the cyan module was negatively correlated with maternal BMI and positively correlated with infant gender, and the tan module was negatively correlated with maternal age. Further investigation of the module stability suggested that the first four modules, including the turquoise, blue, brown, and yellow modules, exhibited much higher stability than the other modules (Fig. 5d).

Further hierarchical clustering indicated that the turquoise module was significantly correlated with birth weight and could clearly separate the FGR and control groups (Fig. 5e). The GO enrichment and signaling pathway analyses suggested that the turquoise module was mainly enriched in osteoclast differentiation and transcriptional misregulation in cancer (Fig. 5f). These findings indicate that the critical module turquoise is significantly correlated with FGR, but further confirmation of these genes in the module by a large sample size will provide more evidence for elucidating FGR.

### The protein-coding genes, lncRNAs, and miRNAs as potential signatures for FGR

**Fig. 6:**
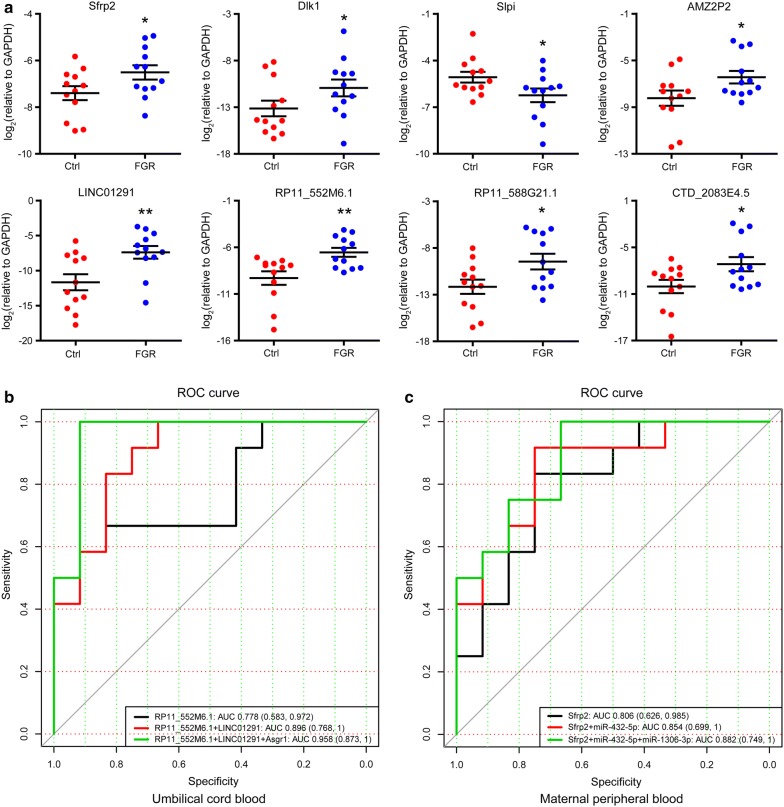
The protein-coding genes, lncRNAs and miRNAs as potential signatures for FGR. **a** Quantitative verification assay of representative genes and lncRNAs in 12 FGR cases and 12 controls (Ctrl) umbilical cord blood samples. The statistical significance was analyzed by Student’s *t*-test. * and ** represented *P* < 0.05 and *P* < 0.01, respectively. **b** The ROC curve for the potential gene signatures including RP11_552M6.1, LINC01291, and Asgr1 in 12 FGR and 12 control umbilical cord blood samples. **c** The ROC curve for the potential predictive markers including Sfrp2, miR-432-5p, and miR-1306-3p in 12 FGR and 12 control maternal peripheral blood samples. ROC, receiver operating characteristic. *AUC* area under the curve

To detect the significance of differentially expressed transcriptome, we detected the expression level of the top-10 up-regulated and top-10 down-regulated molecules for protein coding genes and lncRNAs, six differentially expressed imprinted genes, and 13 differentially expressed miRNAs (Additional file 4: Table S4) in 12 FGR and 12 control umbilical cord blood samples (Additional file 2: Table S2) by quantitative RT-PCR analyses. The representative two imprinted genes (Sfrp2 and Dlk1), Slpi, and five lncRNAs (LINC01291, RP11_552M6.1, RP11_588G21.1, CTD_2083E4.5, and AMZ2P2) were shown in Fig. 6a. Furthermore, we performed a forward stepwise logistic regression analysis, and the ROC plot showed that RP11_552M6.1, LINC01291, and Asgr1 in final model could make the AUC value to 0.958 (Fig. 6b), indicating significantly predictive potential for FGR. To observe the application potential in clinic, we examined the expression level of the differentially expressed molecules in the 12 pairs of FGR case and control maternal peripheral blood samples. The ROC plot showed that the expression pattern of Sfrp2, miR-432-5p, and miR-1306-3p had significantly predictive power (AUC = 0.882) in maternal peripheral blood of FGR (Fig. 6c). These findings suggeste that a cluster of protein-coding genes, lncRNAs, and miRNAs that is critically correlated with FGR and may provide potential signatures of FGR. Further investigation of their diagnostic potential in a large sample size at early stage of pregnancy, and the functional study on significant signatures may improve the understanding of FGR.

## Discussion

Due to the lack of case–control studies investigating fetal-originated umbilical cord blood, the regulatory networks of FGR remain unclear, and the early prediction and diagnosis of FGR are challenging. In this case–control study, we performed a systematic whole-transcriptome profiling of the functional regulatory networks in human FGR case and control umbilical cord blood samples. The differentially expressed whole-transcriptome, imprinted genes, and weighted gene co-expression analyses revealed an enrichment in functional processes and critical modules related to growth, development, and the immune system.

Several studies have demonstrated that maternal factors such as age, BMI, preeclampsia, chronic hypertension, and anemia are associated with FGR [16, 32]. Fetal factors and placental factors, including malformation, infections, and abnormal placental vascular system are also associated with FGR. The measurement of placental biomarkers in maternal blood is a common method used to evaluate placental functions related to pregnancy outcomes [33]. However, these placental molecules are heterogeneous and identified in various locations, such as placental tissue, amniochorionic membranes, amniotic fluid, cord blood and maternal blood [34]. The sample heterogeneity in the placenta and multifactorial nature of FGR especially abnormal fetal growth, should be confirmed by repeated ultrasound. Thus, matching controls to each FGR infant based on these characteristics could eliminate their potential influences on fetal weight and yield more constructive evidences. Our study focused on a case–control study of the entire transcriptome of umbilical cord blood to demonstrate the most core gene networks in FGR. The combination of the core gene expression signatures and ultrasound confirmation could more effectively separate normal small infants from pathological FGR infants, and lead to an effective strategy for FGR intervention or even prevention at an early time-point.

As FGR confers a high risk of increased perinatal, childhood, and adulthood complications, effective screening and treatment procedures are critical for avoiding adverse health outcomes in neonates born with FGR [35]. Here, we used RNA sequencing to assess the entire transcriptome (mRNA, lncRNA, and miRNAs), then uncovered the significant signatures and potential signaling pathways of FGR. It has been reported that FGR may lead to a cardiovascular risk, metabolic problems, and poor neurodevelopmental outcomes in adulthood [36, 37]. Based on system biology and network-based analyses of the differentially expressed whole-transcriptome, this study not only confirmed that the differentially expressed genes of FGR at the whole-transcriptome level are significantly enriched in metabolism and neural and cardiac systems but also elucidated the critical correlation between the differentially expressed transcriptome and immune system diseases, such as Graft-versus-host disease and systemic lupus erythematosus. These findings provide more potential and effective approaches for immune treatment of FGR. Since human umbilical cord blood includes various cell types such as lymphocytes, monocytes, and mesenchymal stem cells [38–40], which may have different effects on the transcriptome profile of protein-coding genes, lncRNAs, and miRNAs. To clearly explore the exact contribution of each cell source of whole blood on RNA profile may provide more specific evidence in the future.

To better capture the inter-gene relationships and define the co-regulatory patterns involved in FGR, we performed WGCNA to explore the systems-level expression changes and clustered the highly-correlated genes into co-expression modules [10, 11]. This study identified five modules related to maternal-infant demographic variables. Interestingly, we found that the turquoise module could clearly separate the FGR group from the controls and was positively correlated with birth weight among the FGR cases. Similarly, we found that the cyan module was negatively correlated with maternal BMI and positively correlated with infant gender, while the tan module was negatively correlated with maternal age. It has been reported that infant gender may affect the placental gene expression and function [41, 42]. Whether infant gender affects the gene expression profile of umbilical cord blood needs further study with a large sample size. And the effects of infant gender on these genes enriched in the tan module also needs further confirmation. To evaluate the utility of these modules in elucidating the molecular underpinnings of FGR, we performed GO and signaling pathway enrichment analyses of each module. These analyses indicated that the significant FGR modules focused on metabolism, immune systems and transcriptional misregulation. However, further studies are warranted to determine whether these gene signatures are relevant to postnatal health, and to provide further mechanistic insight into the consequences of abnormal fetal growth.

Furthermore, to systematically investigate the core regulatory network of FGR at the whole-transcriptome level, we performed an integrated analysis of protein-coding genes, lncRNAs, and miRNAs. By combining the lncRNA-mRNA, miRNA-mRNA, miRNA-lncRNA, and protein–protein interaction (PPI) analyses, we observed 7 *cis*-, 59 *trans*-, 2 miRNA, and 70 PPI regulatory relationships. LncRNAs and protein-coding genes in *cis*-regulatory relationship had a positive PCC, while lncRNAs and *trans*-regulated genes had a negative PCC, which is similar to miRNAs and their targets. It has been reported that imprinted genes can regulate growth and development [31]. Here, we found that the expression levels of six imprinted genes (Col9a3, Dlk1, Fuca1, Lilrb4, Sfrp2, and Ventx) significantly differed between FGR cases and controls. Furthermore, we confirmed the expression level of the top-10 differentially expressed protein-coding genes and lncRNAs, six imprinted genes and 13 miRNAs in umbilical cord blood and maternal peripheral blood. The ROC plots showed that RP11_552M6.1, LINC01291, and Asgr1 in umbilical cord blood, while Sfrp2, miR-432-5p, and miR-1306-3p in the maternal peripheral blood had significantly predictive power for FGR. These findings suggest a cluster of molecular signatures that are potentially diagnostic and predictive markers of FGR.

Since the limited sample size used in this study, the stability of the gene modules defined by the WGCNA has been further investigated by the bootstrap method [28]. In consistent with Shannon et al., the distribution of the Jaccard similarity coefficients of each module between the real data and bootstrapped set suggested that the gene modules with large gene sizes had higher stability than the small sized modules. Furthermore, we confirmed the turquoise, blue, brown, and yellow modules exhibited much higher stability than the other modules as compared with the best-case stability of the random modules in the simulation. Furthermore, considering with the limited sample size in this study, these 95% CI AUCs of logistic regression models were also calculated by 1000 bootstraps. Then, these findings need further investigation of the diagnostic potential of these potential molecules in a large sample size, and their relevance to postnatal health in childhood and adulthood for the consequences of abnormal fetal growth.

## Conclusions

This study comprehensively profiled the transcriptome-wide landscape of human umbilical cord blood, constructed the core gene co-expression network, delineated the critical gene signatures including imprinted genes correlated with FGR, and provided key insight into intrauterine perturbations and candidate signatures of FGR. However, it needs further exploration for the diagnostic significance in a large sample size during early stage of pregnancy, and functional and mechanistic study to provide more evidences for elucidating FGR.

## Supplementary information

**Additional file 1: Table S1.** Characteristics for the five pairs of FGR cases and controls in RNA-sequencing.

**Additional file 2: Table S2.** Characteristics for the 12 pairs of FGR cases and controls in the verification assay.

**Additional file 3: Table S3.** Primer sequences used in qRT-PCR assays.

**Additional file 4: Table S4.** Differentially expressed protein-coding genes, lncRNAs, and miRNAs.

**Additional file 5: Table S5.** GSEA of key GO item enrichment.

**Additional file 6: Table S6.** GSEA of key signaling pathway enrichment.

**Additional file 7: Table S7.** GSEA of key hallmark enrichment.

**Additional file 8: Table S8.** Hierarchical clustering of the 18 modules.

## Data Availability

The RNA sequencing data were deposited in the National Omics Data Encyclopedia (NODE) database (https://www.biosino.org/node) under project ID: OEP000732. The data that support the findings of the current study are available from the corresponding author on reasonable request.

## References

[1] Barker DJ (1995). The Wellcome Foundation Lecture, 1994. Proc Biol Sci.

[2] de Boo HA, Harding JE (2006). The developmental origins of adult disease (Barker) hypothesis. Aust N Z J Obstet Gynaecol.

[3] Gluckman PD, Hanson MA (2004). Living with the past: evolution, development, and patterns of disease. Science.

[4] Miller SL, Huppi PS, Mallard C (2016). The consequences of fetal growth restriction on brain structure and neurodevelopmental outcome. J Physiol.

[5] Crispi F, Bijnens B, Sepulveda-Swatson E (2014). Postsystolic shortening by myocardial deformation imaging as a sign of cardiac adaptation to pressure overload in fetal growth restriction. Circ Cardiovasc Imaging.

[6] Ornoy A (2011). Prenatal origin of obesity and their complications: gestational diabetes, maternal overweight and the paradoxical effects of fetal growth restriction and macrosomia. Reprod Toxicol.

[7] Monier I, Blondel B, Ego A, Kaminiski M, Goffinet F, Zeitlin J (2015). Poor effectiveness of antenatal detection of fetal growth restriction and consequences for obstetric management and neonatal outcomes: a French national study. BJOG.

[8] Longo S, Borghesi A, Tzialla C, Stronati M (2014). IUGR and infections. Early Hum Dev.

[9] Mifsud W, Sebire NJ (2014). Placental pathology in early-onset and late-onset fetal growth restriction. Fetal Diagn Ther.

[10] Zhang B, Horvath S (2005). A general framework for weighted gene co-expression network analysis. Stat Appl Genet Mol Biol.

[11] Langfelder P, Horvath S (2008). WGCNA: an R package for weighted correlation network analysis. BMC Bioinform.

[12] Whitehead CL, McNamara H, Walker SP (2016). Identifying late-onset fetal growth restriction by measuring circulating placental RNA in the maternal blood at 28 weeks’ gestation. Am J Obstet Gynecol.

[13] Tang L, He G, Liu X, Xu W (2017). Progress in the understanding of the etiology and predictability of fetal growth restriction. Reproduction.

[14] Ruchob R, Rutherford JN, Bell AF (2018). A systematic review of placental biomarkers predicting small-for-gestational-age neonates. Biol Res Nurs.

[15] Vayssière C, Sentilhes L, Ego A (2015). Fetal growth restriction and intra-uterine growth restriction: guidelines for clinical practice from the French College of Gynaecologists and Obstetricians. Eur J Obstet Gynecol Reprod Biol.

[16] Frederick IO, Williams MA, Sales AE, Martin DP, Killien M (2008). Pre-pregnancy body mass index, gestational weight gain, and other maternal characteristics in relation to infant birth weight. Matern Child Health J.

[17] Dai L, Deng C, Li Y (2014). Birth weight reference percentiles for Chinese. PLoS ONE.

[18] Weng R, Lu C, Liu X (2018). Long noncoding RNA-1604 orchestrates neural differentiation through the miR-200c/ZEB axis. Stem Cells.

[19] Kim D, Langmead B, Salzberg SL (2015). HISAT: a fast spliced aligner with low memory requirements. Nat Methods.

[20] Wang L, Wang S, Li W (2012). RSeQC: quality control of RNA-seq experiments. Bioinformatics.

[21] Liao Y, Smyth GK, Shi W (2014). featureCounts: an efficient general purpose program for assigning sequence reads to genomic features. Bioinformatics.

[22] Love MI, Huber W, Anders S (2014). Moderated estimation of fold change and dispersion for RNA-seq data with DESeq2. Genome Biol.

[23] Huang DW, Sherman BT, Tan Q (2007). DAVID Bioinformatics Resources: expanded annotation database and novel algorithms to better extract biology from large gene lists. Nucleic Acids Res.

[24] Subramanian A, Tamayo P, Mootha VK (2005). Gene set enrichment analysis: a knowledge-based approach for interpreting genome-wide expression profiles. Proc Natl Acad Sci USA.

[25] Tilford CA, Siemers NO (2009). Gene set enrichment analysis. Methods Mol Biol.

[26] Liberzon A, Subramanian A, Pinchback R, Thorvaldsdóttir H, Tamayo P, Mesirov JP (2011). Molecular signatures database (MSigDB) 3.0. Bioinformatics.

[27] Zhang H, Meltzer P, Davis S (2013). RCircos: an R package for Circos 2D track plots. BMC Bioinform.

[28] Shannon CP, Chen V, Takhar M (2016). SABRE: a method for assessing the stability of gene modules in complex tissues and subject populations. BMC Bioinform.

[29] Cianciulli A, Calvello R, Porro C, Trotta T, Panaro MA (2017). Understanding the role of SOCS signaling in neurodegenerative diseases: current and emerging concepts. Cytokine Growth Factor Rev.

[30] Wang H, Wang J, Xia Y (2017). Defective suppressor of cytokine signaling 1 signaling contributes to the pathogenesis of systemic lupus erythematosus. Front Immunol.

[31] Jirtle RL, Skinner MK (2007). Environmental epigenomics and disease susceptibility. Nat Rev Genet.

[32] Ota E, Ganchimeg T, Morisaki N (2014). Risk factors and adverse perinatal outcomes among term and preterm infants born small-for-gestational-age: secondary analyses of the WHO Multi-Country Survey on Maternal and Newborn Health. PLoS ONE.

[33] Zhong Y, Zhu F, Ding Y (2015). Serum screening in first trimester to predict pre-eclampsia, small for gestational age and preterm delivery: systematic review and meta-analysis. BMC Pregnancy Childbirth.

[34] Heazell AE, Whitworth M, Duley L, Thornton JG (2015). Use of biochemical tests of placental function for improving pregnancy outcome. Cochrane Database Syst Rev.

[35] Barker DJ, Hales CN, Fall CH, Osmond C, Phipps K, Clark PM (1993). Type 2 (non-insulin-dependent) diabetes mellitus, hypertension and hyperlipidaemia (syndrome X): relation to reduced fetal growth. Diabetologia.

[36] Saleem T, Sajjad N, Fatima S, Habib N, Ali SR, Qadir M (2011). Intrauterine growth retardation–small events, big consequences. Ital J Pediatr.

[37] Savchev S, Sanz-Cortes M, Cruz-Martinez R (2013). Neurodevelopmental outcome of full-term small-for-gestational-age infants with normal placental function. Ultrasound Obstet Gynecol.

[38] Broxmeyer HE, Douglas GW, Hangoc G (1989). Human umbilical cord blood as a potential source of transplantable hematopoietic stem/progenitor cells. Proc Natl Acad Sci USA.

[39] Lee OK, Kuo TK, Chen WM, Lee KD, Hsieh SL, Chen TH (2004). Isolation of multipotent mesenchymal stem cells from umbilical cord blood. Blood.

[40] Goodwin HS, Bicknese AR, Chien SN, Boqucki BD, Quinn CO, Wall DA (2001). Multilineage differentiation activity by cells isolated from umbilical cord blood: expression of bone, fat, and neural markers. Biol Blood Marrow Transplant.

[41] Mueller BR, Bale TL (2008). Sex-specific programming of offspring emotionality after stress early in pregnancy. J Neurosci.

[42] Alexander J, Teague AM, Chen J (2018). Offspring sex impacts DNA methylation and gene expression in placentae from women with diabetes during pregnancy. PLoS ONE.

