# Supplementary material for: Whole-transcriptome sequencing uncovers core regulatory modules and gene signatures of human fetal growth restriction
Source: Clin Transl Med. 2020 Jan 28;9:9. doi: 10.1186/s40169-020-0259-0 (PMC6987274; doi:10.1186/s40169-020-0259-0)
Supplement: Supplementary file 1 — Additional file 1: Table S1. Characteristics for the five pairs of FGR cases and controls in RNA-sequencing. [file 40169_2020_259_MOESM1_ESM.docx]

**Table S1. Characteristics for the five pairs of FGR cases and controls in RNA-sequencing.**

| **Sample**  **(ID)** | **Maternal**  **age (years)** | | | **Maternal**  **BMI (kg/m^2^)** | | **Gestational**  **age (weeks)** | | **Infant gender** | | **Birth**  **score** | | **Birth**  **weight (kg)** | |
| --- | --- | --- | --- | --- | --- | --- | --- | --- | --- | --- | --- | --- | --- |
| **FGR (N = 5)** | |  |  | |  | |  | |  | |  | |  |
| FGR-1 | 38 | | | 27.4 | | 39.7 | | Female | | 10 | | 2.490 | |
| FGR-2 | 35 | | | 26.0 | | 40.4 | | Male | | 10 | | 2.535 | |
| FGR-3 | 29 | | | 24.1 | | 37.3 | | Female | | 10 | | 2.185 | |
| FGR-4 | 29 | | | 24.3 | | 37.7 | | Female | | 10 | | 2.435 | |
| FGR-5 | 28 | | | 24.7 | | 37.4 | | Female | | 10 | | 1.575 | |
| Mean | 31.8 | | | 25.3 | | 38.5 | | / | | / | | 2.244 | |
| Std | 4.44 | | | 1.37 | | 1.45 | | / | | / | | 0.398 | |
| **Ctrl (N = 5)** | |  |  | |  | |  | |  | |  | |  |
| FGR-1c | 32 | | | 26.2 | | 39.4 | | Female | | 10 | | 3.565 | |
| FGR-2c | 35 | | | 27.8 | | 39.9 | | Female | | 10 | | 3.630 | |
| FGR-3c | 33 | | | 22.8 | | 37.7 | | Male | | 10 | | 2.740 | |
| FGR-4c | 30 | | | 22.2 | | 37.7 | | Male | | 10 | | 3.150 | |
| FGR-5c | 43 | | | 27.2 | | 38.1 | | Female | | 10 | | 2.915 | |
| Mean | 34.6 | | | 25.3 | | 38.6 | | / | | / | | 3.200 | |
| Std | 5.03 | | | 2.58 | | 1.01 | | / | | / | | 0.392 | |
| *P* value | 0.46 | | | 0.95 | | 0.80 | | 1.00 | | / | | 0.0025 | |

FGR, fetal growth restriction; Ctrl, appropriate for gestational age; BMI, body mass index. RNA sequencing was performed in a case-control study with the umbilical cord blood of FGR cases (FGR1-5) and paired controls (FGR1c-5c). The statistical significance of the difference between FGR and control group was determined by Student's *t* test for continues variables (maternal age, BMI, gestational age, and birth weight) and Fisher's exact test for categorical variables (infant gender). It indicates significant when the *P* value < 0.05.
