# Supplementary material for: Whole-transcriptome sequencing uncovers core regulatory modules and gene signatures of human fetal growth restriction
Source: Clin Transl Med. 2020 Jan 28;9:9. doi: 10.1186/s40169-020-0259-0 (PMC6987274; doi:10.1186/s40169-020-0259-0)
Supplement: Supplementary file 2 — Additional file 2: Table S2. Characteristics for the 12 pairs of FGR cases and controls in the verification assay. [file 40169_2020_259_MOESM2_ESM.docx]

**Table S2. Characteristics for the 12 pairs of FGR cases and controls in the verification assay.**

| **Sample**  **(ID)** | **Maternal**  **age (years)** | | | **Maternal**  **BMI (kg/m^2^)** | | **Gestational**  **age (weeks)** | | **Infant gender** | | **Birth**  **score** | **Birth**  **weight (kg)** | | |
| --- | --- | --- | --- | --- | --- | --- | --- | --- | --- | --- | --- | --- | --- |
| **FGR (N = 12)** | |  |  | |  | |  | |  | | |  |  |
| FGR-6 | 34 | | | 27.3 | | 38.7 | | Female | | 10 | 2.585 | | |
| FGR-7 | 36 | | | 26.8 | | 36.6 | | Male | | 9 | 1.910 | | |
| FGR-8 | 30 | | | 27.0 | | 37.3 | | Female | | 9-10 | 2.450 | | |
| FGR-9 | 31 | | | 22.7 | | 38.4 | | Female | | 9-10 | 2.540 | | |
| FGR-10 | 34 | | | 31.3 | | 36.7 | | Female | | 9-10 | 2.225 | | |
| FGR-11 | 25 | | | 24.2 | | 39.7 | | Female | | 10 | 2.500 | | |
| FGR-12 | 32 | | | 23.6 | | 35.4 | | Female | | 10 | 2.005 | | |
| FGR-13 | 27 | | | 26.0 | | 39.9 | | Female | | 10 | 2.485 | | |
| FGR-14 | 32 | | | 30.0 | | 36.1 | | Male | | 9 | 1.920 | | |
| FGR-15 | 26 | | | 26.9 | | 35.1 | | Female | | 9 | 1.375 | | |
| FGR-16 | 29 | | | 24.5 | | 38.0 | | Female | | 9 | 2.500 | | |
| FGR-17 | 36 | | | 22.5 | | 39.9 | | Male | | 9 | 2.350 | | |
| Mean | 31.0 | | | 26.1 | | 37.7 | | / | | / | 2.237 | | |
| Std | 3.72 | | | 2.74 | | 1.71 | | / | | / | 0.366 | | |
| **Ctrl (N = 12)** | |  |  | |  | |  | |  | | |  |  |
| FGR-6c | 35 | | | 25.2 | | 38.0 | | Male | | 10 | 3.240 | | |
| FGR-7c | 28 | | | 26.5 | | 36.6 | | Male | | 9-10 | 3.030 | | |
| FGR-8c | 36 | | | 28.0 | | 37.9 | | Female | | 9 | 3.120 | | |
| FGR-9c | 39 | | | 25.5 | | 39.0 | | Male | | 10 | 3.070 | | |
| FGR-10c | 27 | | | 30.5 | | 38.6 | | Female | | 10 | 2.925 | | |
| FGR-11c | 30 | | | 23.4 | | 40.0 | | Female | | 9 | 3.595 | | |
| FGR-12c | 34 | | | 23.7 | | 39.9 | | Female | | 10 | 3.225 | | |
| FGR-13c | 30 | | | 26.7 | | 39.6 | | Male | | 9 | 3.270 | | |
| FGR-14c | 33 | | | 30.5 | | 36.0 | | Male | | 9-10 | 2.960 | | |
| FGR-15c | 35 | | | 25.7 | | 37.6 | | Male | | 10 | 2.775 | | |
| FGR-16c | 29 | | | 25.0 | | 37.9 | | Female | | 9 | 3.350 | | |
| FGR-17c | 32 | | | 22.7 | | 39.7 | | Male | | 9 | 3.690 | | |
| Mean | 32.3 | | | 26.1 | | 38.4 | | / | | / | 3.188 | | |
| Std | 3.63 | | | 2.52 | | 1.30 | | / | | / | 0.268 | | |
| *P* value | 0.45 | | | 0.96 | | 0.24 | | 0.21 | | / | <0.0001 | | |

FGR, fetal growth restriction; Ctrl, appropriate for gestational age; BMI, body mass index. The significantly correlated protein-coding genes, lncRNAs, and miRNAs as obtained in RNA-sequencing were quantitatively verified in the umbilical cord blood and maternal peripheral cord blood of FGR6-17 and FGR6c-17c. It indicates significant when *P* value < 0.05.
