# Supplementary material for: Whole-transcriptome sequencing uncovers core regulatory modules and gene signatures of human fetal growth restriction
Source: Clin Transl Med. 2020 Jan 28;9:9. doi: 10.1186/s40169-020-0259-0 (PMC6987274; doi:10.1186/s40169-020-0259-0)
Supplement: Supplementary file 3 — Additional file 3: Table S3. Primer sequences used in qRT-PCR assays. [file 40169_2020_259_MOESM3_ESM.docx]

**Table S3. Primer sequences used in qRT-PCR assays.**

| **Gene symbols** | **Forward Primer** | **Reverse Primer** |
| --- | --- | --- |
| Gapdh | ATGACATCAAGAAGGTGGTG | CATACCAGGAAATGAGCTTG |
| Begain | GCTGTCCTACACCACACACA | GATCCTCTGCAGTGCCATGT |
| Asgr1 | GCTACTGGTTCTCTCGCTCC | CCCATCCAGGTGTTCACAGG |
| F8 | TCCGTTTGCACCCAACTCAT | TTTGAAGGAGACCAGGTGGC |
| Slpi | CCTGGATCCTGTTGACACCC | ACAGGGGAAACGCAGGATTT |
| FolR2 | GCACCACAAGACAAAGCCAG | AAAGTTGTACAGGCGGGAGG |
| FolR3 | GCAAGCGCCACTTTATCCAG | TTTGCAGGTGTAGGAGGTGC |
| Fuca1 | GGCCTCATCGGGATTTGGTT | GGTCGTACAGCTCTGGCATT |
| Lilrb4 | GCCCAAGAACAAGGCCAGAT | GTCACAAGAGGACTCGGCAG |
| Ventx | CAGCACCACCAGTACCTGAG | TCCTGCATTTGCCGTTTGTG |
| Sfrp2 | ACAACGACATAATGGAAACGCT | GCCACAGCACCGATTTCTTC |
| Dlk1 | GCGAGGATGACAATGTTTGC | TCTATCACAGAGCTCCCCGTC |
| Areg | GGTGTGGGGAAAAGTCCATGA | CTTTCGTTCCTCAGCTTCTCCT |
| Dok6 | GAGTGCAGCGGGAACAGAAT | CTGAGAGGCCACATCACCAG |
| Klrc2 | TCCCCGAATACAAGAACGCA | AGGCCAGCAAACTCTCTTCC |
| Agtr1 | AGCTATGGAATACCGCTGGC | GGCTACAAGCATTGTGCGTC |
| Klrd1 | TCCCAGTATCTACTCTGACTGCT | GAAGCACAGAGATGCCGACT |
| LINC01291 | GACCAGCAGCATGTGTTTCC | TTTACTGGGTGGTGCAGTCA |
| RP11-552M6.1 | CTGACGACCCTTGGCTATGT | TTCTCAGGAATAGCCTGCTGC |
| RP11_588G21.1 | ACCATGGGGCTGATGTTTGT | GCCAGTCAGGCTGGTTAGTT |
| AMZ2P2 | GGCTTGAGAGCAAACCTCCT | ATGACACAGGCGTCTTCAGG |
| CTD_2083E4.5 | ATTCAAGCGACTTGCAGCG | CCGGGATCATTTCTCTTGATGTC |
| RP11-506K6.4 | GTGAGATGGACTCTTGGGGC | AGGAACCTCTGCGTCAACAC |
| RP11-121C2.2 | CAGACAAGCACAGGCAGGTA | GCCAGTGTCCTTGTATCCCC |
